# Realized genetic gain for yield and yield attributes in groundnut breeding at ICRISAT from an ERA trial

**DOI:** 10.3389/fpls.2025.1640041

**Published:** 2025-09-17

**Authors:** Partha Pratim Behera, Dnyaneshwar Deshmukh, Anil Kumar Vemula, Kiranmayee Bangaru, Rachana Bagudam, Anurag Mathew, Ashutosh Purohit, Aparna Vishnumolakala, Ankush P. Wankhade, Safinaaz Kounain, Nehru Guguloth, Murali T. Variath, Mukhthambica Kurva, Gopi Potupureddi, Surya Muragesan, Anitha Raman, Janila Pasupuleti

**Affiliations:** Accelerated Crop Improvement (ACI)- Groundnut Breeding, International Crops Research Institute for the Semi-Arid Tropics (ICRISAT), Hyderabad, India

**Keywords:** ERA trials, groundnut, realized genetic gain, pod yield, shelling outturn percentage, hundred kernel weight

## Abstract

**Introduction:**

Groundnut or peanut (*Arachis hypogaea* L.) is an important food and oilseed crop with a global production of >50 m t from ~34 m ha. The ICRISAT groundnut breeding program, established in 1976, has significantly contributed to varietal development, resulting in the release of >240 varieties in 39 countries. Estimating realized genetic gain (RGG) in a breeding program helps to measure the progress made for agronomic traits and identify gaps to guide the breeding strategy.

**Materials and methods:**

This study was conducted to estimate realized genetic gain using an Elite Replicated Agronomic (ERA) trial, with five ERA trials representing three product concepts across market types and maturity durations. These trials included improved germplasm developed over a span of 15–20 years at ICRISAT and were evaluated for three key traits: pod yield (PY), 100 seed weight (HSW), and shelling outturn (SP).

**Results and discussion:**

Among these, PY and HSW exhibited high repeatability and genetic advance as the percentage of mean, whereas SP showed lower values. Realized genetic gain varied from 8.37 kg ha^-^¹ year^−1^ (0.48%) to 54.85 kg ha^-^¹ year^−1^ (3.91%) for PY. The Spanish Bunch germplasm recorded a higher realized GG of 46.45 kg ha^-^¹ year^−1^ (2.95%) for pod yield, compared to the Virginia Bunch germplasm with a marginal gain of 5.97 kg ha^-^¹ year^−1^ (0.23%). Higher RGG is realized in medium-duration and late-maturing germplasm with 27.1 kg ha^-^¹ year^−1^ (1.62%) and 25.32 kg ha^-^¹ year^−1^ (1.52%), respectively, while realized GG in early-maturing germplasm was 8.37 kg ha^-^¹ year^−1^ (0.5%). Among the traits, RGG was the highest for PY across all the trials. Higher RGG for PY and HSW was observed during the rainy season as compared to the post-rainy season, while SP showed a decline. This study helps breeders to optimize selection methods and design breeding strategies to enhance realized genetic gain for SP across two market types and three maturity durations. The study suggests a need for breeding strategies to enhance the rate of RGG for PY in early-maturing germplasm.

## 1 Introduction

Groundnut (*Arachis hypogaea* L.) is an important food and oilseed crop that contributes to food and nutritional security. Its kernels are a valuable source of protein, oil, dietary fiber, vitamins, antioxidants, and micronutrients (Abadya et al., 2021). In addition to nutritional value, groundnut also contributes to environmental sustainability through biological nitrogen fixation, thus reducing greenhouse gas emissions. Despite its importance, groundnut yields remain low in regions like Africa (~1 ton ha^−1^) and India due to lack of access to quality seed of improved cultivars, suboptimal inputs and crop husbandry, and biotic and abiotic stress, compared to the high yield potential of ~4 tons ha^−1^ observed in the USA and China (FAOSTAT, 2023). Climate change further exacerbates the situation because of erratic rainfall patterns, rising temperatures, and an increase in the frequency of extreme weather events, all of which adversely affect groundnut productivity (Abadya et al., 2021; Janila et al., 2016a). Abiotic (drought, heat stress, salinity, and nutrient-deficient soils) and biotic stresses (foliar fungal diseases, soil-borne pathogens, and insect pests) significantly reduce pod yield and quality (Janila et al., 2016a; Daudi et al., 2021; Meena et al., 2025). Increasing productivity per unit area in an environmentally sustainable manner ensures food and economic security for millions of farmers and consumers worldwide (Hu et al., 2025; Varshney et al., 2020). To address these challenges, it is essential to accelerate the development of groundnut cultivars with improved pod yield and quality, enhanced tolerance to abiotic and biotic stresses, and efficient water use, thereby ensuring sustainable production under climate change scenarios. Accelerated cultivar delivery can be achieved through an efficient breeding program aimed at increasing the rate of genetic gain.

The efficiency of a plant breeding program is evaluated using well-defined key performance indicators (KPIs), which are mainly categorized into three components: design (market segmentation and product profiling), engineering (population improvement and product development), and delivery (product commercialization and variety renewal) (Sangh et al., 2025). These KPIs include metrics such as the ratio of farmer-adopted varieties to the number of crosses, the cost–benefit ratio of newly released varieties, and the response to selection over time. For these breeding gains to translate into higher adoption rates, it is imperative that the product profiles align closely with the needs of various stakeholders, including farmers, value-chain participants, consumers, and funders (Sangh et al., 2025; Janila et al., 2013). Such alignment ensures that the breeding targets reflect the practical requirements and preferences of end users, leading to widespread acceptance and use of the newly developed varieties. Among these indicators, genetic gain (GG) is a high-level KPI for assessing the overall breeding progress. It is defined as the improvement achieved in the desired traits over successive breeding cycles by artificial selection. GG is critical for driving variety turnover and ensuring that new cultivars meet evolving agricultural challenges and market demands (Kadirimangalam et al., 2022). It serves as a benchmark for comparing different crossing, evaluation, and selection strategies, either through real experiments or simulations (Cobb et al., 2013; Walsh and Lynch, 2018; Faux et al., 2016). The expected genetic gain in a breeding program is derived from the breeder’s equation, which includes key parameters such as heritability, selection differential, selection intensity, and genetic variance (Burrows, 1972; Walsh and Lynch, 2018). However, the expected genetic gain guides selection strategies, and it does not reflect the actual progress achieved under field conditions. In contrast, realized genetic gain (RGG) relies on phenotypic data collected from germplasm evaluated across different stages of testing, such as early generation trials, advanced yield trials, and farmer-managed environments (Covarrubias-Pazaran, 2020). Thus, RGG provides a more accurate improvement achieved in target traits over time and serves as a critical indicator for assessing the long-term impact and effectiveness of the breeding pipeline (Mrode, 2014; Rutkoski, 2019a, 2019). Such an assessment provides critical information on the health of the breeding program and guides the design of the breeding program to enhance the rate of genetic gain for target traits.

RGG is calculated either using historical data (Mackay et al., 2011; Piepho et al., 2014) or by evaluating different varieties developed or released over a time period in Elite Replicated Agronomic (ERA) trials (Masuka et al., 2017; Seck et al., 2023; Delgado et al., 2024; Tarekegne et al., 2024). In ERA trials, elite breeding lines or cultivars released in different years are evaluated under uniform field conditions across locations and years. RGG using ERA trials is estimated by regressing genotype means for the trait studied on the year of release, resulting in unbiased estimates of genetic gain. These trials minimize the confounding effects from differences in agronomic management practices or climate variability (Rutkoski, 2019a, 2019). However, limitations include a restricted number of selected cultivars representing each ERA, and they are evaluated only in a few environments, which makes the results less applicable to other conditions (Seck et al., 2023). Despite the limitations, ERA trials have been successfully deployed in several crops to measure the breeding progress, such as rice (Venkatanagappa et al., 2021), maize (Tarekegne et al., 2024), winter wheat (Boehm et al., 2023), spring wheat (Gerard et al., 2024), cassava (Delgado et al., 2024), and potato (Sood et al., 2022). For instance, Venkatanagappa et al. (2021) reported a notable RGG of 0.70% per year (23 kg ha^-^¹ annually), though this gain slightly declined to 0.62% when adjusted for maturity in IRRI-developed rice varieties over a 50-year period. Similarly, Tarekegne et al. (2024) documented annual grain yield increases of 118, 63, 46, and 61 kg ha^-^¹ under optimum, low nitrogen, managed drought, and random stress conditions, respectively, in maize, spanning from 2000 to 2018. These findings underline the efficacy of ERA trials as a robust tool for measuring genetic improvement and guiding future breeding strategies.

The International Crops Research Institute for the Semi-Arid Tropics (ICRISAT) and national programs in China, India, the USA, Africa, and Australia maintain long-term groundnut breeding programs; however, RGG is not estimated by these programs. One of the primary reasons is the lack of available historical data from multi-environment trials, and historically, the investment in groundnut breeding is low compared to major cereals (Janila et al., 2013; Pandey et al., 2020). At ICRISAT and in some national programs, although historical data are available, inconsistencies in the check continuum prevent its use for estimating RGG. The check continuum is essential for estimating RGG in crop breeding programs because it provides a consistent benchmark for comparing the performance of breeding lines over time. ERA trials are a viable option when historical data are lacking, but they are resource-intensive since a new dataset must be generated specifically for these trials. Moreover, establishing an ERA trial requires seed multiplication of varieties released over the past two or more decades, which is challenging due to the low seed multiplication ratio of groundnut. This is the first documented estimate of realized genetic gain of a groundnut breeding program.

Various studies have demonstrated how monitoring genetic trends in crop cultivars facilitates the identification of successful breeding practices and guides future programs (Covarrubias-Pazaran, 2020). At ICRISAT, the groundnut breeding program is focused on achieving measurable genetic gain for key traits outlined in the product concepts while ensuring the maintenance of genetic diversity. The present study attempts to estimate the RGG within the ICRISAT groundnut breeding program to assess the progress made, identify gaps, and design informed future breeding strategies.

The objective of the study is to estimate the RGG for three key yield traits, viz., pod yield (PY), shelling percentage (SP), and 100-seed weight (HSW), which is done in a comprehensive manner using five distinct ERA trials. It examines RGG trends across two seasons (rainy or post-rainy) and three product concepts that are determined by maturity duration and market types (Spanish and Virginia). Product concepts (PCs) are strategic, forward-looking definitions of the types of groundnut varieties (products) that the ICRISAT groundnut breeding program aims to develop. They guide breeding priorities by aligning genetic improvement efforts with market needs, farming systems, and environmental challenges. PC-1 is early maturing with ~90 to 100 days duration in Spanish-type varieties for home consumption and oil extraction, PC-2 is medium-maturing varieties with ~120 days duration in both Spanish and Virginia types to meet home consumptions needs as well as food and oil industry needs, and PC-3 is late-maturing varieties with >120 days duration in both Spanish and Virginia types for confectionary uses. In groundnut, Spanish (*A. hypogaea* subsp. *fastigiata* var. *vulgaris*) and Virginia (*A. hypogaea* subsp. *hypogaea* var. *hypogaea*) are two of the major botanical and market types, each with distinct characteristics that influence their use in different agro-ecologies and markets. The growth habit of Spanish types is erect and compact and generally early, while Virginia types are late with a spreading or semi-spreading growth habit; however, artificial hybridization between Spanish and Virginia types in the breeding programs produced Virginia types with a compact growth habit and reduced maturity duration. The insight from this study aims to support the development of high-yielding and climate-resilient groundnut varieties that meet the needs of the market and consumers.

## 2 Materials and methods

### 2.1 Trials, genotypes, and experimental years

Five ERA trials representing three PCs, viz., PC-1, PC-2, and PC-3, of two market types of Spanish Bunch (SB) and Virginia Bunch (VB) were conducted to evaluate three key yield-related traits, namely, PY, SP, and HSW. The details of these trials are as follows:

1. Elite Yield Trial Genetic Gain-Product Concept-1 (EYTGG-PC-1): The trial evaluated a set of 42 early-maturing genotypes (40 test genotypes and 2 checks) released over a span of 20 years (1993–2017) (**Supplementary Table 1**). The trial was conducted in a 7 × 6 lattice design over four seasons (post-rainy 2017–2018, rainy 2018, post-rainy 2018–2019, and rainy 2019) with two replications at ICRISAT in Patancheru.
2. Elite Yield Trial Genetic Gain-Spanish Bunch-Product Concept-2 (EYTGG-SB-PC-2): This trial comprised 56 medium-maturing SB genotypes from studies conducted over a 21-year period, from 1988 to 2017 (**Supplementary Table 2**; **Table 1**). The trial was conducted in an 8 × 7 alpha lattice design with two replications at ICRISAT, Patancheru, across four seasons (post-rainy 2017–2018, rainy 2018, post-rainy 2018–2019, and rainy 2019).
3. Elite Yield Trial Genetic Gain-Virginia Bunch-Product Concept-2 (EYTGG-VB-PC-2): The trial involved 25 medium-maturing VB genotypes (23 test genotypes and 2 checks) from studies over a 15-year period (1994–2017) (**Supplementary Table 3**; **Table 1**). The experiment was conducted in a 5 × 5 alpha lattice design with two replications at ICRISAT, Patancheru, across four seasons (post-rainy 2017–2018, rainy 2018, post-rainy 2018–2019, and Rainy 2019).
4. Elite Yield Trial Genetic Gain-Spanish Bunch-Product Concept-3 (EYTGG-SB-PC-3): A total of 28 (26 test genotypes and 2 checks) late-maturing SB genotypes selected across 16 years (1995–2014) based on their HSW and high PY (**Supplementary Table 4**; **Table 1**) were included in this trial. These genotypes were tested in three seasons (post-rainy 2017–2018, rainy 2018, and post-rainy 2018–2019) in a randomized complete block design (RCBD) with two replications at ICRISAT, Patancheru.
5. Elite Yield Trial Genetic Gain-Virginia Bunch-Product Concept-3 (EYTGG-VB-PC-3): This trial was designed to evaluate 16 (15 test entries and 1 check) late-maturing VB genotypes from studies conducted over a 12-year period (1994–2014) (**Supplementary Table 5**; **Table 1**). This experiment was laid out in an RCBD with two replications and was conducted over three seasons (post-rainy 2017–2018, rainy 2018, and post-rainy 2018–2019) at ICRISAT, Patancheru.

**Table 1:** Detailed description of ERA trials conducted at ICRISAT.

S. no.	ERA trial name	Maturity	Species	Season	No. of seasons	Experimental design	Entries (genotypes + checks)	Checks	Duration of the study to include genotypes in these trials
1	Elite Yield Trial Genetic Gain-Product Concept-1 (EYTGG-PC-1)	Early		PR 2017–2018	4	7 × 6 (alpha lattice)	40 + 2	JL 24, TMV 2	20 (1993–2017)
				R 2018					
				PR 2018–2019					
				R 2019					
2	Elite Yield Trial Genetic Gain-SB-Product Concept-2 (EYTGG-SB-PC-2)	Medium	Spanish	PR 2017–2018	4	8 × 7 (alpha lattice)	56 + 0	No checks	21 (1987–2017)
				R 2018					
				PR 2018–2019					
				R 2019					
3	Elite Yield Trial Genetic Gain-VB-Product Concept-2 (EYTGG-VB-PC-2)	Medium	Virginia	PR 2017–2018	4	5 × 5 (alpha lattice)	23 + 2	ICGV 86699, ICGV 87846	15 (1988–2017)
				R 2018					
				PR 2018–2019					
				R 2019					
4	Elite Yield Trial Genetic Gain-SB-Product Concept-3 (EYTGG-SB-PC-3)	Late	Spanish	PR 2017–2018	3	RCBD	26 + 2	TKE-19-A, TPG 41	16 (1995–2014)
				R 2018					
				PR 2018–2019					
5	Elite Yield Trial Genetic Gain-VB-Product Concept-3 (EYTGG-VB-PC-3)	Late	Virginia	PR 2017–2018	3	RCBD	15 + 1	ICGV 86564	12 (1988–2014)
				R 2018					
				PR 2018–2019					

RCBD, randomized complete block design; R, Rainy season; PR, Post-rainy season.

### 2.2 Statistical analysis

For each ERA trial, a joint analysis across 2 years was conducted using a linear mixed model that considers genotypes as fixed and years, replications within years, and blocks within replications as random, which were fitted for the measured traits using SAS software version 9.4 (SAS Institute Inc, 2023) (Equation 1).


(1) $$\begin{array}{l} \text{RCBD}:y_{ijk}=\mu +g_{i}+m_{j}+r_{jk}+gm_{ij}+\epsilon_{ijk}\\ \text{Alpha Lattice}:y_{ijk}=\mu +g_{i}+m_{j}+r_{jk}+b{\left(r\right)}_{jkl}+gm_{ij}+\epsilon_{ijkl} \end{array}$$

where $Y_{ijk}\;$ is the trait observation of the *k*th replicate of the *i*th genotype in the *j*th year, μ is the overall mean, $g_{i}\;$ is the effect of the *i*th genotype, $m_{j}\;$ is the effect of the *j*th year, $r_{jk}$ is the effect of the *k*th replicate in the *j*th year, b(r)jkl is the effect of l_th_ incomplete block within the k_th_ replicate of the j_th_ environment, $gm_{ij}\;$ is the genotype × year interaction, $\epsilon_{ijk},\;$ is the residual associated with the *k*th replicate of the *i*th genotype in the *j*th year, and $\epsilon_{ijkl},\;$ is the residual associated with the *l*th block within the *r*th replicate of the *i*th genotype in the *j*th year.

To assess genetic trends in varieties, means data were used as the response variable in the linear regression model,


$$y_{ij}=\;a+\;bd_{i}+\epsilon_{i}$$

where $y_{ij}$ is the adjusted mean yield of *i*th line first tested in the *j*th year, *a* is the intercept, $d_{i}$ is the year of first testing in the trial, b is the linear regression coefficient (rate of genetic gain per year), and $\epsilon_{i}$ is the residual variance plus deviation from the regression model. A genetic gain was considered statistically significant when the probability associated with the regression coefficient was less than 0.05 (*P* < 0.05).

The variance components from this model were used to calculate the genetic parameters using the following equations:


$$\text{Genetic variance}:\;GV=\;\sigma_{g}^{2}$$

$$\text{Phenotypic variance}:PV=\sigma_{p}=\;\sigma_{g}^{2}+\frac{\sigma_{gm}^{2}}{m}+\;\frac{\sigma_{\epsilon }^{2}}{rm}$$

$$\;\;\text{Broad sense heritability}:H=\;\frac{\sigma_{g}^{2}}{\sigma_{g}^{2}+\frac{\sigma_{gm}^{2}}{m}+\;\frac{\sigma_{\epsilon }^{2}}{rm}}$$

$$\text{Coefficient of variation}:\;CV\%=\;\frac{\sigma_{\epsilon }}{\bar{µ}}\;\times \;100$$

Broad-sense heritability is the proportion of phenotypic variance that is due to the genotype. A high heritability indicates that the trait is less influenced by the environment. It separates genotypic variance from the environment-induced variance. $\sigma_{g}^{2}$ is the genotypic variance, $\sigma_{gm}^{2}\;$ is the genotype × year interaction variance, $\sigma_{\epsilon }^{2}\;$ is the weighted average residual variance of the individual years weighted by the respective error degrees of freedom, $\sigma_{p}^{2}\;$ is the phenotypic variance, m is the number of years, r is the number of replications within a trial, and i   is the selection intensity at 5% selection proportion. *µ* is the mean value. Best linear unbiased estimates (BLUEs) of entries estimated using Eq. (1) were then regressed on the year of cultivar release. The slope of the regression represents the rate of change of gain per unit time.

## 3 Results

**Figure 1:**
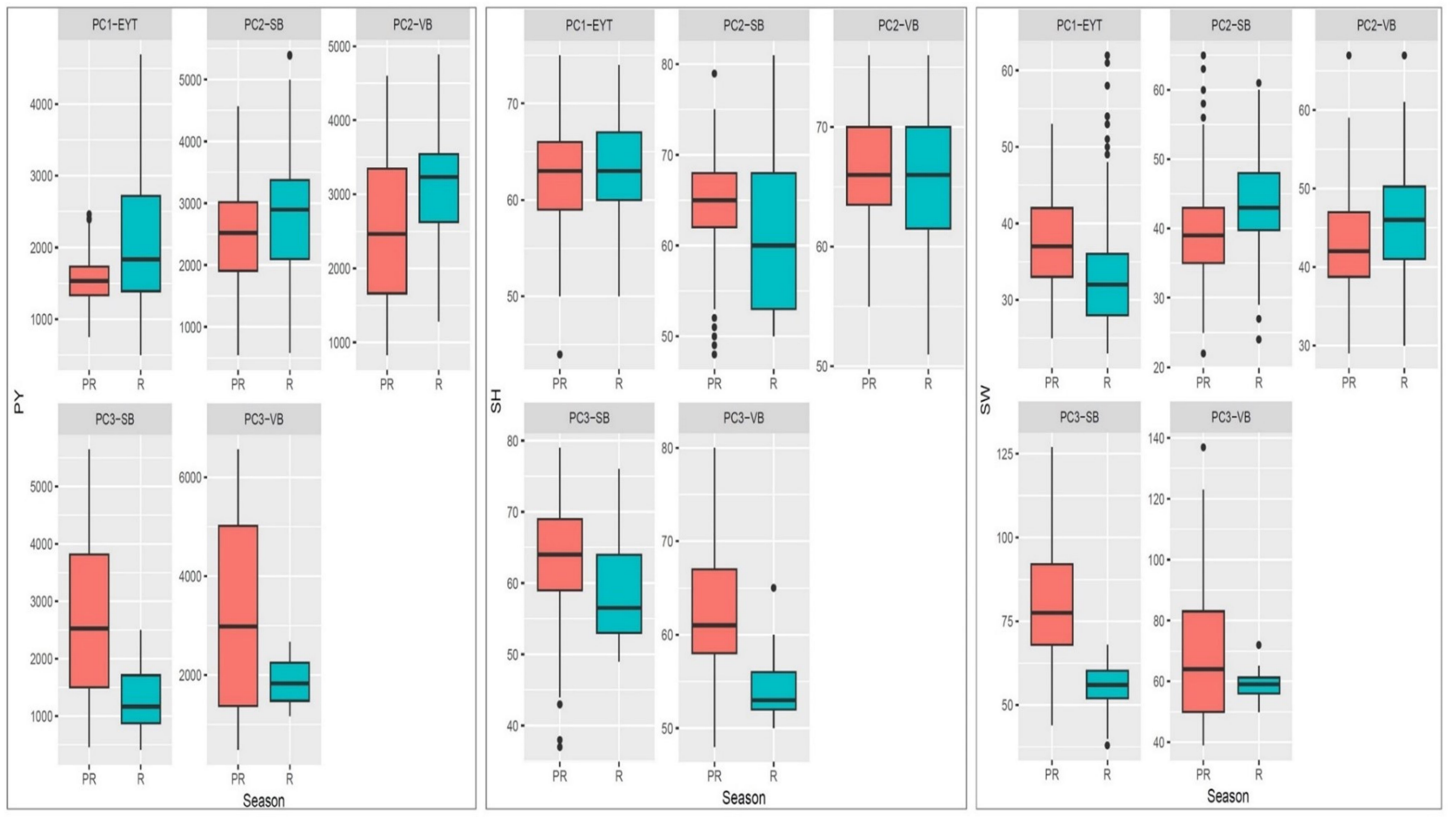
Box plots showing variation in performances for pod yield (PY), shelling percentage (SP), and 100 seed weight (HSW) across seasons and trials in ICRISAT groundnut lines, where PC1-YET: EYTGG-PC-1; PC2-SB: EYTGG-SB-PC-2; PC2-VB: EYTGG-SB-PC-2; PC3-SB: EYTGG-SB-PC-3; PC3-VB: EYTGG-SB-PC-3. R- Rainy season, PR-Post-rainy season.

**Figure 2:**
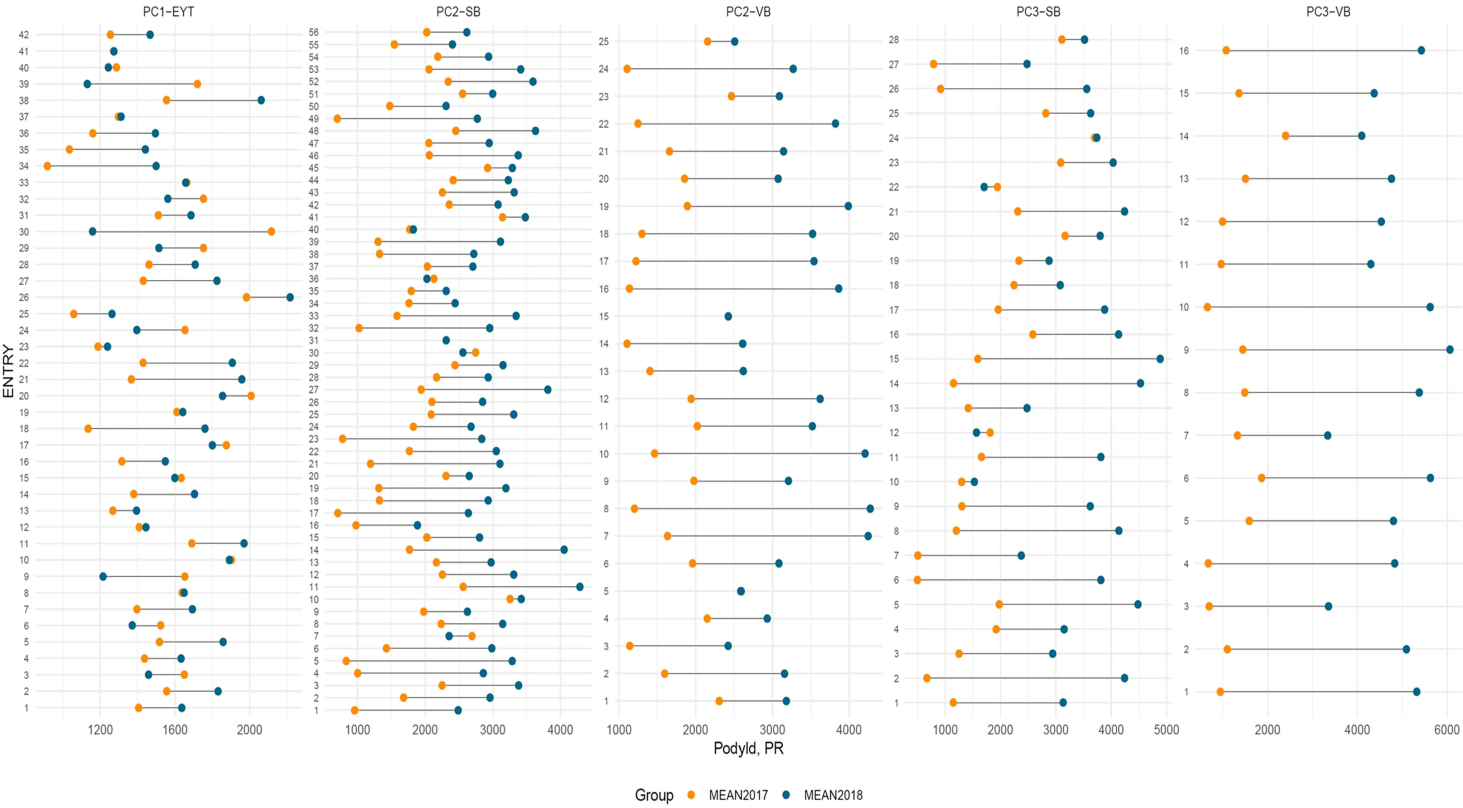
Lollipop plot showing variation in performances for pod yield (PY) across seasons and trials in ICRISAT groundnut lines, where PC1-YET: EYTGG-PC-1; PC2-SB: EYTGG-SB-PC-2; PC2-VB: EYTGG-SB-PC-2; PC3-SB: EYTGG-SB-PC-3; PC3-VB: EYTGG-SB-PC-3. R- Rainy season, PR-Post-rainy season.

In both medium- and late-maturing germplasm, variability for PY was higher during the post-rainy season than in the rainy season. Differences in the distribution for PY between the two seasons were prominent in most of the trials and for HSW in the late-maturing VB trial (EYTGG-VB-PC-3) (**Figures 1**, **2**). Significant genetic variation and year effect for PY, SP, and HSW were observed in the medium-maturing SB germplasm (EYTGG-SB-PC-2). Significant genotype × year interactions (G × Y) for PY and genetic variation for HSW were observed in the early-maturing germplasm (EYTGG-PC-1).

Likelihood ratio tests identified significant genotype × year interactions (G × Y) in the rainy season trials for all traits in both early- and medium-maturing germplasm, where the trials were conducted in both seasons, while the late-maturing germplasm was tested for only 1 year (**Table 2**). Meanwhile, the year effect was non-significant in most trial types, and the G × Y interactions were significant for PY, HSW, and SP. In the post-rainy trials, significant G × Y was observed for PY in all trials except in the late-maturing VB germplasm (EYTGG-VB-PC-3); HSW exhibited significant G × Y in the medium-maturing germplasm of both market types (EYTGG-SB-PC-2 and EYTGG-VB-PC-2), while SP showed significant G × Y in the late-maturing germplasm of both market types (EYTGG-SB-PC-3 and EYTGG-VB-PC-3) (**Table 3**).

**Table 2:** Variance components and genetic parameters of the post-rainy season trials across the 2 years.

Trial	Maturity/market type	Trait	$\sigma_{m}^{2}$	$\sigma_{g}^{2}$	$\sigma_{gm}^{2}$	$\sigma_{\epsilon }^{2}$	CV%	Mean	H
EYTGG-PC-1	Early	SP	0.80 (NS)	4.26	3.90 (NS)	2017: 10.57	6.88	62.21	0.46
						2018: 26.04			
EYTGG-PC-1	Early	HSW	0	12.17**	4.90 (NS)	2017: 8.59	11.12	37.62	0.69
						2018: 26.41			
EYTGG-PC-1	Early	PY	2,660.42 (NS)	18,789	22,177*	2017: 39,086	14.02	1,550.81	0.51
						2018: 55,472			
EYTGG-SB-PC-2	Medium/Spanish	SP	2.78 (NS)	7.78**	1.43 (NS)	2017: 11.37	5.78	64.56	0.71
						2018: 16.48			
EYTGG-SB-PC-2	Medium/Spanish	HSW	9.71,(NS)	20.43**	6.78**	2017: 8.76	8.02	39.52	0.81
						2018: 11.31			
EYTGG-SB-PC-2	Medium/Spanish	PY	575,321**	90,617*	144,523**	2017: 140,596	14.28	2,414.01	0.51
						2018: 97,090			
EYTGG-VB-PC-2	Medium/Virginia	SP	0.20 (NS)	0	11.60*	2017: 18.41	5.86	66.22	0
						2018: 11.71			
EYTGG-VB-PC-2	Medium/Virginia	HSW	0.00	0	22.41**	2017: 19.75	10.13	43.44	0
						2018: 18.98			
EYTGG-VB-PC-2	Medium/Virginia	PY	1,205,700*	0	271,849**	2017: 125,502	13.79	2,498.57	0
						2018: 112,050			
EYTGG-SB-PC-3	Late/Spanish	SP	0.00	1.27	11.39**	2017: 11.39	10.53	62.22	0.08
						2018: 74.52			
EYTGG-SB-PC-3	Late/Spanish	HSW	158.65 (NS)	0	50.44 (NS)	2017: 21.15	16.12	80.38	0
						2018: 314.54			
EYTGG-SB-PC-3	Late/Spanish	PY	1,266,600*	120,116	481,836**	2017: 174,568	19.87	2,593.26	0.3
						2018: 356,462			
EYTGG-VB-PC-3	Late/Virginia	SP	13.07 (NS)	9.55	13.30**	2017: 13.30	7.94	62.33	0.58
						2018: 35.74			
EYTGG-VB-PC-3	Late/Virginia	HSW	576.28 (NS)	15.61	32.77 (NS)	2017: 23.25	12.56	71.51	0.33
						2018: 138.1			
EYTGG-VB-PC-3	Late/Virginia	PY	6,333,500**	1,911	261,754 (NS)	2017: 33,711	16.9	3,042.76	0.01
						2018: 495,201			

$\sigma_{g}^{2}$ , genetic variance; $\sigma_{m}^{2}$ , year variance; $\sigma_{gm}^{2}$ , genotype × year variance; $\sigma_{\epsilon }^{2}$ , residual variance; CV, coefficient of variation; H, heritability; SP, shelling percentage; HSW, hundred seed weight; PY, pod yield. * P<0.05; ** P<0.01, NS- non significant.

**Table 3:** Variance components and genetic parameters of the rainy season trials across the 2 years.

Trial	Maturity/market type	Trait	$\sigma_{m}^{2}$	$\sigma_{g}^{2}$	$\sigma_{gm}^{2}$	$\sigma_{\epsilon }^{2}$	CV%	Mean	H
EYTGG-PC-1	Early	SP	11.03*	1.59	11.15**	2017: 8.63	4.6	62.87	0.19
						2018: 8.07			
EYTGG-PC-1	Early	HSW	53.86**	7.32	19.51**	2017: 4.40	8.49	33.36	0.43
						2018: 11.62			
EYTGG-PC-1	Early	PY	676,900*	22357	357,098**	2017: 62,290	12.45	2,050.35	0.11
						2018: 68,050			
EYTGG-SB-PC-2	Medium/Spanish	SP	90.88**	0.28	5.09*	2017: 8.19	6.23	60.98	0.06
						2018: 20.66			
EYTGG-SB-PC-2	Medium/Spanish	HSW	30.95*	2.19	14.09**	2017: 12.55	9.76	43.09	0.17
						2018: 22.85			
EYTGG-SB-PC-2	Medium/Spanish	PY	515,746**	27590**	253,906*	2017: 155,085	14.62	2,746.47	0.64
						2018: 167,399			
EYTGG-VB-PC-2	Medium/Virginia	SP	3.32	0	11.22*	2017: 22.55	6.47	65.73	0
						2018: 13.60			
EYTGG-VB-PC-2	Medium/Virginia	HSW	4.42	0	16.96	2017: 54.74	13.45	45.56	0
						2018: 20.38			
EYTGG-VB-PC-2	Medium/Virginia	PY	50,271	73,495	186,691**	2017: 285,569	14.94	3,087.69	0.36
						2018: 140,010			
EYTGG-SB-PC-3	Late/Spanish	SP		0		65.88	13.14	61.77	0
EYTGG-SB-PC-3	Late/Spanish	HSW		5.39		37.54	10.77	56.89	0.3
EYTGG-SB-PC-3	Late/Spanish	PY		235,594**		61,603	19.44	1,276.75	0.92
EYTGG-VB-PC-3	Late/Virginia	SP		6.67**		4.03	3.7	54.26	0.83
EYTGG-VB-PC-3	Late/Virginia	HSW		9.61		11.23	5.49	61	0.72
EYTGG-VB-PC-3	Late/Virginia	PY		49,340		131,160	19.51	1,856.58	0.53

$\sigma_{g}^{2}$ , genetic variance; $\sigma_{m}^{2}$ , year variance; $\sigma_{gm}^{2}$ , genotype × year variance; $\sigma_{\epsilon }^{2}$ , residual variance; CV, coefficient of variation; H, heritability; SP, shelling percentage; HSW, hundred seed weight; PY, pod yield. * P<0.05; ** P<0.01.

The genetic variability parameters are presented in **Tables 2**, **3**. Among the rainy season trials, medium-maturing SB germplasm (EYTGG-SB-PC-2) exhibited the highest broad-sense heritability (50% to 80%) for PY, SP, and HSW. High heritability for PY was recorded in medium-maturing SB and VB germplasm (EYTGG-SB-PC-2 and EYTGG-VB-PC-2) and late-maturing SB and VB germplasm (EYTGG-SB-PC-3 and EYTGG-VB-PC-3) in the rainy season. In contrast, in the post-rainy season, low heritability was observed for PY in medium-maturing VB germplasm (EYTGG-VB-PC-2) and late-maturing SB and VB germplasm (EYTGG-SB-PC-3 and EYTGG-VB-PC-3). The heritability of SP was medium to high in early-maturing and medium-maturing SB germplasm (EYTGG-SB-PC-2) during the post-rainy season. Late-maturing VB germplasm (EYTGG-VB-PC-3) recorded high heritability for SP in both the rainy and post-rainy seasons. The CV was high for PY across all the seasons.

Medium-maturing VB germplasm (EYTGG-VB-PC-2) exhibited the highest mean pod yield of 2,793 kg ha^−1^ across seasons, followed by medium-maturing SB germplasm with a mean of 2,580 kg ha^−1^ (**Table 4**; **Figure 1**). Notably, late-maturing VB germplasm showed a low rainy season PY (1,857 kg ha^−1^) but the highest post-rainy PY of 3,043 kg ha^−1^. For HSW, the late-maturing VB germplasm (EYTGG-VB-PC-3) recorded an average of 66 g, while the SB germplasm (EYTGG-SB-PC-3) recorded an average of 54 g across the seasons. Medium-maturing VB germplasm (EYTGG-VB-PC-2) outperformed others with an average SP of 66% across the seasons.

**Table 4:** Trend analysis of ERA trials of the Elite Yield Trials for genetic gain.

Trial name	Year of trial conducted		Traits (units)	Trait BLUE mean			Annual rate of RGG		
	Rainy	Post-rainy		Rainy	Post-rainy	Across seasons	Rainy	Post-rainy	Across seasons
EYTGG-PC-1	2018, 2019	2017, 2018	PY (kg ha^-^¹ year^−1^)	2,050.35	1,550.81	1,800.58	22.7	−5.97	8.37 (0.48%)
			HSW (g year^−1^)	33.36	37.62	35.49	0.148	0.157	0.15 (0.44%)
			SP (% year^−1^)	62.87	62.21	62.54	0.0727	0.045	0.06 (0.097%)
EYTGG-SB-PC-2	2018, 2019	2017, 2018	PY (kg ha^-^¹ year^−1^)	2,746.47	2,414.01	2,580.24	41.4	34.7	38.05 (2%)
			HSW (g year^−1^)	43.09	39.52	41.31	0.068	−0.0229	0.023 (0.055%)
			SP (% year^−1^)	60.98	64.56	62.77	−0.0385	0.0768	0.019 (0.02%)
EYTGG-VB-PC-2	2018, 2019	2017, 2018	PY (kg ha^-^¹ year^−1^)	3,087.69	2,498.57	2,793.13	18.3	14	16.15 (0.64%)
			HSW (g year^−1^)	45.56	43.44	44.50	−0.11	−0.182	−0.15 (−0.31%)
			SP (% year^−1^)	65.73	66.22	65.98	0.147	−0.0045	0.07 (0.11%)
EYTGG-SB-PC-3	2018	2017, 2018	PY (kg ha^-^¹ year^−1^)	638.38	2,593.26	1,615.82	54.3	55.4	54.85 (3.91%)
			HSW (g year^−1^)	28.45	80.38	54.41	0.0259	0.23	0.13 (0.19%)
			SP (% year^−1^)	30.89	62.22	46.55	−0.338	−0.0462	−0.19 (−0.29%)
EYTGG-VB-PC-3	2018	2017, 2018	PY (kg ha^−^¹ year^−1^)	1,856.58	3,042.75	2,449.67	−10.3	1.89	−4.21 (−0.17%)
			HSW (g year^-1^)	61.00	71.5	66.25	0.235	0.248	0.24 (0.38%)
			SP (% year^−1^)	54.26	62.32	58.29	−0.136	−0.0842	−0.11 (−0.18%)

BLUE, best linear unbiased estimate; RGG, realized genetic gain; SP, shelling percentage; HSW, hundred seed weight; PY, pod yield.

**Figure 3:**
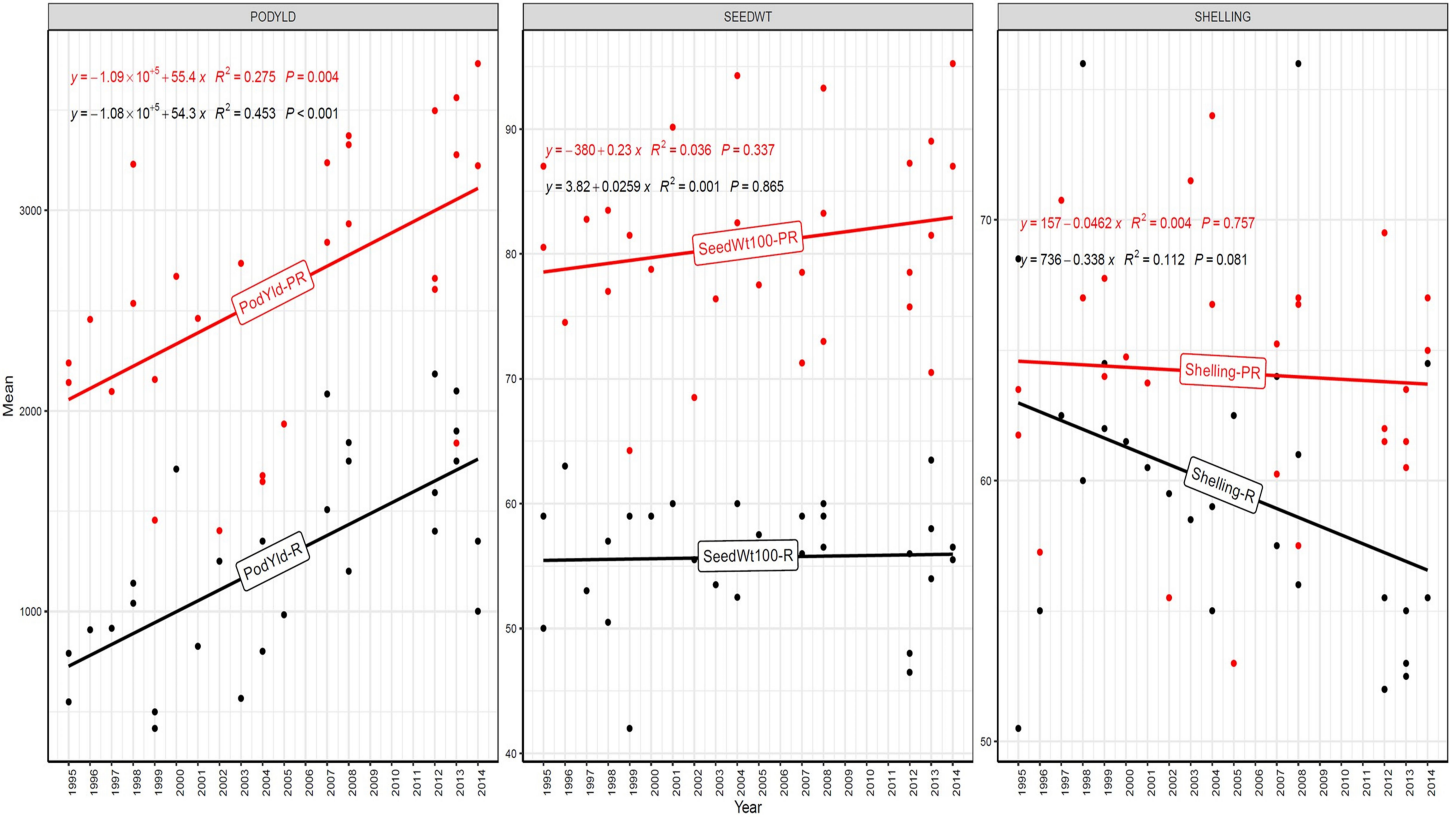
Genetic gain trend analysis in EYTGG-PC-1 ERA trials of ICRISAT groundnut lines, where pod yield (kg ha^-^¹ year^−1^)—PODYLD/PodYld; hundred seed weight (g year^−1^)—SEEDWT/Seedwt100; shelling percentage (% year^−1^)—SHELLING/Shelling; R—rainy season; PR—post-rainy season. Probability associated with the regression coefficient (*P*), if it is less than 0.05 (*P* < 0.05)—annual rate of RGG for the respective trait is significant.

**Figure 4:**
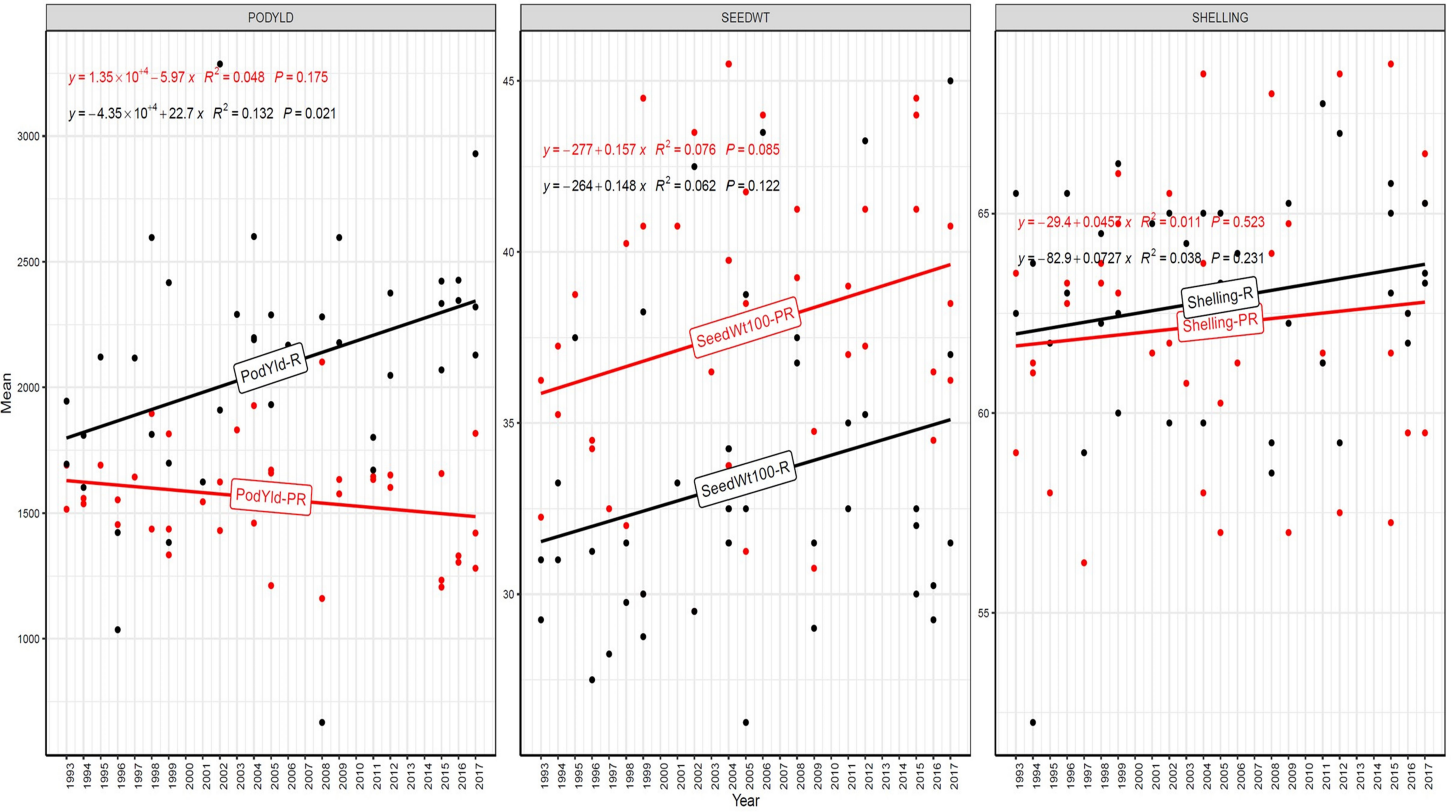
Genetic gain trend analysis in EYTGG-SB-PC-2 ERA trial of ICRISAT groundnut lines, where pod yield (kg ha^-^¹ year^−1^)—PODYLD/PodYld; hundred seed weight (g year^−1^)—SEEDWT/Seedwt100; shelling percentage (% year^−1^)—SHELLING/Shelling; R—rainy season; PR—post-rainy season. Probability associated with the regression coefficient (*P*), if it is less than 0.05 (*P* < 0.05)—annual rate of RGG for the respective trait is significant.

**Figure 5:**
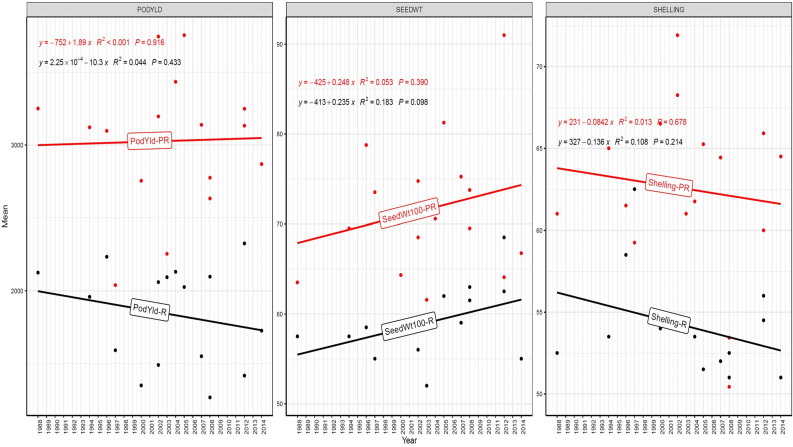
Genetic gain trend analysis in EYTGG-VB-PC-2 ERA trial of ICRISAT groundnut lines, where pod yield (kg ha^-^¹ year^−1^)—PODYLD/PodYld; hundred seed weight (g year^−1^)—SEEDWT/Seedwt100; shelling percentage (% year^−1^)—SHELLING/Shelling; R—rainy season; PR—post-rainy season. Probability associated with the regression coefficient (*P*), if it is less than 0.05 (*P* < 0.05)—annual rate of RGG for the respective trait is significant.

**Figure 6:**
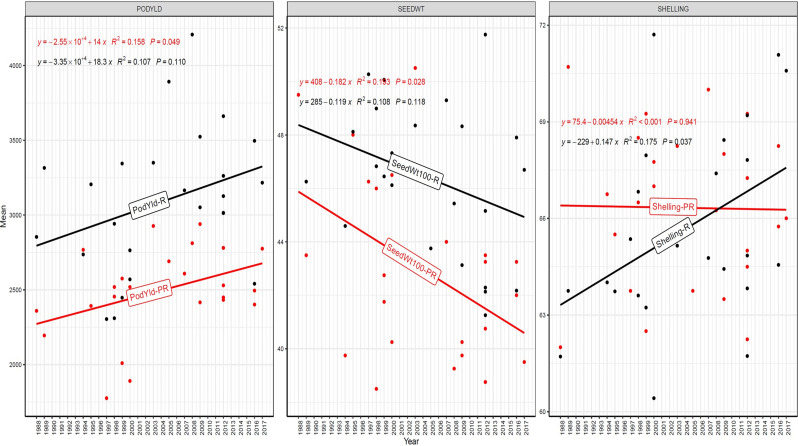
Genetic gain trend analysis in EYTGG-SB-PC-3 ERA trial of ICRISAT groundnut lines, where pod yield (kg ha^-^¹ year^−1^)—PODYLD/PodYld; hundred seed weight (g year^−1^)—SEEDWT/Seedwt100; shelling percentage (% year^−1^)—SHELLING/Shelling; R—rainy season; PR—post-rainy season. Probability associated with the regression coefficient (*P*), if it is less than 0.05 (*P* < 0.05)—annual rate of RGG for the respective trait is significant.

**Figure 7:**
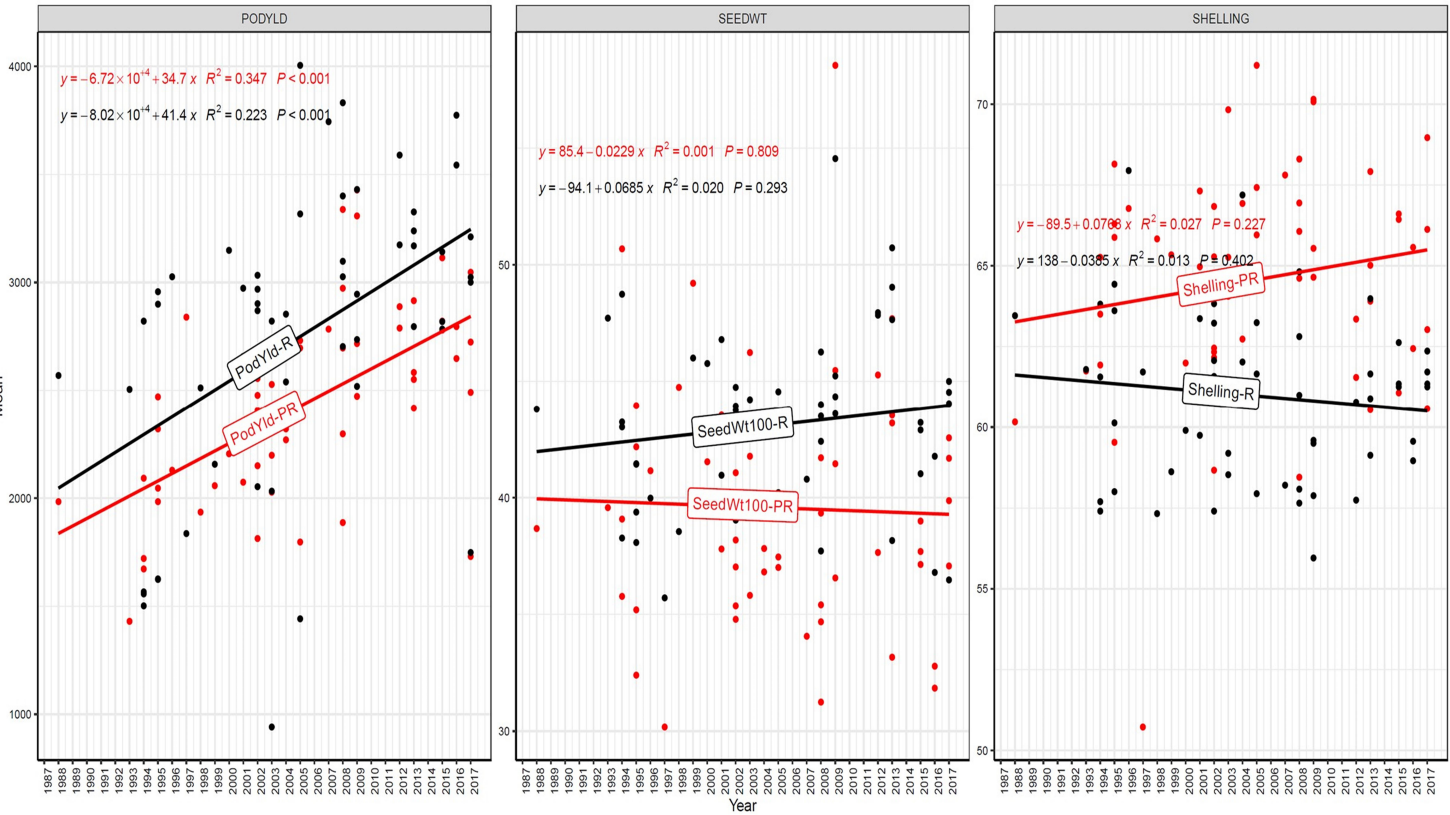
Genetic gain trend analysis in EYTGG-VB-PC-3 ERA trial of ICRISAT groundnut lines, where pod yield (kg ha^-^¹ year^−1^)—PODYLD/PodYld; hundred seed weight (g year^−1^)—SEEDWT/Seedwt100; shelling percentage (% year^−1^)—SHELLING/Shelling; R—rainy season; PR—post-rainy season. Probability associated with the regression coefficient (*P*), if it is less than 0.05 (*P* < 0.05)—annual rate of RGG for the respective trait is significant.

The trend in RGG was analyzed for the rate of change of RGG per year for the three traits, viz., PY, HSW, and SP, across five different studies (EYTGG-PC-1, EYTGG-SB-PC-2, EYTGG-VB-PC-2, EYTGG-SB-PC-3, and EYTGG-VB-PC-3) in both rainy and post-rainy seasons (**Table 4**; **Figures 3**–**7**).

In early-maturing germplasm (EYTGG-PC-1), the RGG of 22.7 kg ha^-^¹ year^-^¹ for PY was higher in the rainy season than in the post-rainy season (−5.97 kg ha^-^¹ year^-^¹), and the same trend was also observed in SP; however, an opposite trend was found for HSW. In medium-maturing VB and SB germplasm (EYTGG-SB-PC-2, EYTGG-VB-PC-2), the RGGs of PY and HSW were found to be higher in the rainy season than in the post-rainy season, but the post-rainy season RGG was higher for SP. Among the medium-duration germplasm, SB germplasm (EYTGG-SB-PC-2) recorded the highest PY gains in the rainy season (41.4 kg ha^-^¹ year^-^¹), while VB germplasm (EYTGG-VB-PC-2) followed a similar trend with lower gains. In late-maturing germplasm, a higher rate of change of RGG was observed in the post-rainy season for all the traits in both SB and VB germplasm, and the rate of change of RGG was higher for PY in late-duration SB germplasm, while it was higher for SP and HSW in VB germplasm. Late-duration SB germplasm exhibited the highest gains overall for all the traits, particularly in the post-rainy season for pod yield with a gain of 55.4 kg ha^-^¹ year^-^¹.

Comparing RGGs across both the rainy and post-rainy seasons, the highest RGG for PY was observed in late-duration SB germplasm (EYTGG-SB-PC-3) [54.85 kg ha^-^¹ year^-^¹ (3.91%)], followed by EYTGG-SB-PC-2 [38.05 kg ha^-^¹ year^-^¹ (2%)], with the lowest in EYTGG-VB-PC-3 [−4.21 kg ha^-^¹ year^-^¹ (−0.17%)]. The rate of change of RGG for PY was up to 54.85 (3.91% per annum) and was in a positive direction in all trials except in early-maturing germplasm (EYTGG-PC-1) in the post-rainy season and late-maturing VB germplasm (EYTGG-VB-PC-3) in the rainy season. For HSW, late-maturing VB germplasm (EYTGG-VB-PC-3) showed the highest RGG [0.24 g year^-^¹ (0.38%)], followed by early-maturing germplasm (EYTGG-PC-1) [0.15 g year^-^¹ (0.44%)], while medium-maturing VB germplasm (EYTGG-VB-PC-2) had the lowest [−0.15 g year^-^¹ (−0.31%)]. For SP, the highest RGG was in medium-maturing VB germplasm (EYTGG-VB-PC-2) [0.07% year^-^¹ (0.11%)], with EYTGG-PC-1 at [0.06% year^-^¹ (0.097%)], and the lowest in late-maturing SB germplasm (EYTGG-SB-PC-3) [−0.19% year^-^¹ (−0.29%)]. The gain per year achieved for HSW was less than 1 g in all five trial types and was negative in both the rainy and post-rainy seasons of medium-duration VB germplasm (**Table 4**). SP showed minimal or negative gains across trials, while the positive gains were small (0.04% to 0.17%). However, medium-duration germplasm achieved the highest genetic gain, with SB germplasm outperforming VB for all the traits. The rainy season favored genetic gain in early- and medium-maturing germplasm, while the post-rainy season was more beneficial for late-maturing germplasm.

## 4 Discussion

Realized GG is an important metric in crop breeding, providing an empirical measure of the genetic improvement achieved over time through selection and breeding practices. It offers insights into the effectiveness of breeding programs, enabling breeders to assess the progress toward desired traits such as yield, disease resistance, and stress tolerance (Rutkoski et al., 2015). ERA trials have been used by crop breeding programs to estimate RGG, as these trials provide unbiased estimates of GG by avoiding differences in agronomic management or climate variability (Rutkoski, 2019a, 2019) and ensuring good connectivity across various cohorts. ERA trials offer valuable insights into the genetic gains achieved through crop breeding programs; however, they have limitations like the need for additional resources and the evaluation of a few lines representing each ERA trial. ERA trials were used to estimate the RGG in different crop species, such as rice (Venkatanagappa et al., 2021), maize (Tarekegne et al., 2024), winter wheat (Boehm et al., 2023), spring wheat (Gerard et al., 2024), cassava (Delgado et al., 2024), and potato (Sood et al., 2022). Realized genetic gain estimated employing ERA trials measures genetic progress using varieties released in different years to represent time periods and includes a representative sample from each cohort. Expected genetic gain, on the other hand, refers to the prediction of the actual change in phenotype that occurs due to the genetic changes brought about by different selection strategies (Fellahi et al., 2018). It is an *a priori* estimate of the genetic gain from a breeding scheme (Seck et al., 2023) and can be used to guide choices on the number of crosses, cycle duration, or the intensity of selection, but it is not used to make complex decisions of crop breeding programs. Expected genetic gain is estimated using data from a single trial or a single generation.

The ICRISAT groundnut breeding program, initiated in 1976, focused on improving pod yield, drought tolerance, early maturity, and foliar fungal disease resistance. As the program evolved, groundnut varieties for confectionary uses with bold-size kernels were also developed. Wild diploid derivatives of *A. cardenassi* were utilized to improve foliar fungal disease resistance (Bertioli et al., 2021), and elite resistant germplasm was shared globally to develop several disease-resistant cultivars. ICRISAT-bred lines were tested, and over 240 varieties were released in 39 different countries and used extensively as parents in the national breeding programs in Africa and Asia. Recently, the first high oleic acid (HOA) groundnut cultivars, ICGV 15083 (Girnar 4) and ICGV 15090 (Girnar 5) with ~80% oleic acid content, were released for cultivation in India. Despite such breeding efforts and progress, genetic enhancement for key traits in groundnut is constrained owing to low heritability, G × E interactions, narrow genetic base, and limited phenotypic precision (Dwivedi et al., 2003; Pasupuleti and Nigam, 2013; Yu and Chen, 2024). Moreover, the genetic base of cultivated groundnut is narrow, limiting access to novel alleles, while the use of wild relatives is constrained by linkage drag and cross-incompatibility. The key target trait, PY, in groundnut is complex, and the low-heritability trait is strongly affected by G × E interactions, which reduces selection accuracy (Radhakrishnan et al., 2022). Additionally, the subterranean nature of groundnut pods poses practical challenges in phenotyping and trait improvement.

To accelerate cultivar development, the ICRISAT groundnut breeding program aims to achieve an increased rate of genetic gain for target traits while simultaneously improving operational and resource efficiency. The current groundnut breeding program at ICRISAT is a demand-led breeding guided by product concepts, which is developed based on market analysis that encompasses both the end-user demand and the needs of the production environment. As ICRISAT’s breeding program aims to accelerate the delivery of climate-resilient groundnut varieties, therefore, assessing the program’s current performance is essential to inform the future design and direction of the breeding strategy. Estimating RGG provides insights into understanding the effectiveness of breeding strategies and identifying gaps for improvement in a breeding program. By evaluating phenotypic data from representative germplasm across multiple environments and time periods, RGG helps in refining breeding strategies and optimizing resource allocation (Piepho et al., 2008; Mrode, 2014; Rutkoski, 2019b). Its utilization ensures that breeding programs remain responsive to changing environmental conditions and market demands, ultimately contributing to sustainable agricultural development.

The present study is aimed at estimating RGG as a measure of breeding progress for three key yield-related traits, PY, SP, and HSW, that can guide future groundnut breeding strategy at ICRISAT. ERA trials were conducted to assess the RGG in two market (or botanical) types, Spanish Bunch and Virginia Bunch, across three maturity durations (early, medium, and late corresponding to three product concepts, PC-1, PC-2, and PC-3, respectively). Elite groundnut lines developed over 15 to 20 years’ duration (1987–2017) were selected and systematically evaluated to assess the RGG for PY, HSW, and SP using ERA trials. Environmental variance was a major contributor to the phenotypic expression of target traits, with highly significant effects observed for PY, HSW, and SP. Significant genotype × year interactions (G × Y) were observed for PY, indicating that the performance of genotypes varied across the years and seasons. The significant role of the environment in determining PY and SP is evident from the differential performances of elite lines in two different seasons. This suggests the need to identify genotypes with specific adaptation to harness the genetic potential for these traits. The significant variability observed in traits across trials highlights opportunities for fine-tuning breeding strategies to improve adaptation in specific environmental conditions (Mousavi-Derazmahalleh et al., 2019; Patil et al., 2014).

In the rainy season, high heritability for PY suggests the potential to improve pod yield in medium- and late-maturing germplasm, while heritability for PY is low in early-maturing germplasm, indicating the limitation in improving PY in early-maturing germplasm. Late-maturing VB germplasm showed high heritability for SP in both seasons. This indicates that this trait is strongly influenced by genetic components, but selection is ineffective due to the presence of non-additive gene effects or significant effects of environment on trait expression and, therefore, needs further testing (Janila et al., 2016a; Pokhrel et al., 2025). The medium-maturing VB germplasm showed zero or low heritability for all chosen traits. This can be attributed to the high G × Y and year effects.

### 4.1 RGG is variable across the rainy and post-rainy seasons for PY, SP, and HSW

In the rainy season, the RGG for PY, SP, and HSW was higher in early- and medium-maturing germplasm, while in long-duration germplasm, the RGG was high in the post-rainy season as the late-maturing germplasm took advantage of irrigation. Moreover, this variation reflects differences in phenological adaptation, resource use, and environmental interactions. Early- and medium-maturing germplasm benefit from shorter growth cycles that align with abundant rainfall, enabling efficient water and nutrient use and drought escape (Rao et al., 2012; Desmae et al., 2019). On the other hand, long-duration genotypes are more suited to the controlled irrigation and extended growing period available in the post-rainy season. These genotypes require more time to achieve their full yield potential and may struggle under the waterlogged or disease-prone conditions often associated with the rainy season (Janila et al., 2013; Pokhrel et al., 2025). In the post-rainy season, a stable environment, minimal disease pressure, and better management of agronomic inputs allow long-duration genotypes to perform optimally, leading to higher genetic gains. These findings emphasize the complex relationship between environmental factors such as moisture availability, temperature, and photoperiod with trait expression and genetic performance in groundnut. Supporting this, Hajjarpoor et al. (2021) found that medium-duration genotypes excelled in the rainy season, due to better water and nutrient use, while Raza et al. (2024) reported superior stability and yield in long-duration types, during the post-rainy season. Based on maturity duration, Venkatanagappa et al. (2021) documented an impressive annual genetic gain of 0.70% (equivalent to 23 kg ha^-^¹) in IRRI-developed rice varieties over five decades. However, when adjusted for maturity, this gain experienced a modest decline to 0.62%, underscoring the dynamic challenges in sustaining genetic progress. The differential RGG across seasons highlights the variable performance of genotypes under different environmental conditions, suggesting season-specific breeding strategies to optimize genetic gains for each trait.

### 4.2 Scope to improve pod yield in early-maturing groundnut germplasm

Early-maturing groundnut varieties are essential for enhancing climate resilience and integrating into crop rotations across diverse cropping systems; however, the RGG suggests limited progress in PY within this germplasm. In groundnut, pod filling and pod maturation duration determine the crop duration, wherein the early-maturing types have the shortest pod filling and maturation duration compared to medium- and late-maturing germplasm. To enhance PY in early-maturing germplasm, selecting traits such as early vigor is crucial. Early vigor promotes rapid establishment and vigorous growth, which supports increased photosynthate production during the initial growth stages (Pasupuleti and Nigam, 2013; Sharma et al., 2024). Additionally, traits that facilitate efficient and rapid translocation of photosynthates from the source (leaves) to sink organs (pods) are essential for maximizing yield potential. Breeding strategies incorporating selection for early vigor, combined with physiological and morphological traits that promote efficient translocation, can lead to the development of early-maturing germplasm with higher pod yields (Parmar et al., 2022).

### 4.3 Spanish types realized a higher rate of RGG for pod yield

In our study, notable genetic gains in pod yield were achieved in Spanish germplasm, reflecting steady progress in the ICRISAT groundnut breeding program. High RGG of over 2% was realized for pod yield; RGG of 54.85 kg ha^-^¹ year^-^¹ (3.91%) was observed in late-maturing SB germplasm, and RGG of 38.05 kg ha^-^¹ year^-^¹ (2%) was observed in medium-maturing SB germplasm on groundnut. This indicates that, on average, the pod yield of newly developed groundnut varieties in these two categories is increasing by >2% per year due to genetic improvement achieved through breeding. It reflects the effectiveness of the ICRISAT breeding program, specifically, how well it is selecting and advancing superior genotypes over time. In winter wheat collected from 1959 to 2021 (over 63 years), significant positive relative RGG was reported for yield with an increase of 39.2 kg ha^−1^ year^−1^ (0.67%) (Boehm et al., 2023).

Spanish-type germplasm has remarkable phenotypic plasticity, enabling them to thrive across diverse environments and consistently express favorable traits such as higher PY even under suboptimal conditions (Janila et al., 2013; Kadiyala et al., 2021; Dey et al., 2024). Breeding programs have prioritized Spanish type for their adaptability and economic value in semiarid regions, focusing on traits like PY, HSW, and oil content. This targeted selection has driven rapid accumulation of favorable alleles, leading to accelerated GG compared to Virginia types (Bohra et al., 2020; Chen et al., 2024; Gerard et al., 2024). Moreover, the efficiency of Spanish types in utilizing water and nutrients drives greater yield gains per unit input, particularly in resource-limited environments (Janila et al., 2016b; Akchaya et al., 2025). Intensively selected for yield and stress resilience, Spanish types, despite a narrower genetic base, harbor a higher frequency of alleles linked to seed weight and drought tolerance (Muhammad et al., 2020; Chen et al., 2024). Their reduced G × E interactions further enhance selection precision and breeding efficiency. In contrast, Virginia types, with longer growing seasons and more G × E variability, show comparatively lower gains across environments (Gelaye and Luo, 2024; Dey et al., 2024).

### 4.4 Groundnut breeding must focus on improving shelling percentage across the maturity groups

Shelling percentage or outturn (SP) in groundnut is a key market trait that determines the market price of the commodity. The stagnation observed in SP from the study highlights a critical gap in the improvement of this key market trait that necessitates deeper investigation. Improving SP in groundnut breeding programs needs an accurate phenotyping approach and a targeted breeding approach, as SP showed low RGG across all trials in the study. Recently, ICRISAT has standardized and deployed a computer tomography (CT) technique to obtain unbiased and precise estimates of SP (Trapp, 2022). These advancements in high-throughput phenotyping ensure a more reliable and accurate trait measurement. Previously, the breeding programs measured SP either manually or mechanically, shelling the pods and weighing the pods and kernels, which is labor-intensive and prone to measurement errors and bias (Bhat et al., 2021; Motagi et al., 2022). Conducting genotype-by-environment interaction studies can refine the understanding of genetic potential, enabling breeders to identify genotypes with stable and desirable SP (Pandey et al., 2020). Emphasizing traits like pod structure and seed-to-shell ratio in early-generation selection may further enhance long-term RGG for SP.

### 4.5 Limitations of the ERA trials and the study

ERA trials provide valuable insights into RGG; however, they come with several limitations. The major constraint is the lack of uniformity in trial design, management practices, and environmental conditions across years, which can confound true genetic progress with non-genetic effects. Additionally, the heterogeneity of test entries, which were originally developed and released for diverse agroecological zones, poses challenges when such trials are conducted at a single location across seasons. Moreover, in such cases, genotype × environment interactions may not be adequately captured, potentially introducing bias in the assessment of genetic trends and limiting the accuracy of genetic gain estimation (Atlin et al., 2000; Yan, 2021). In this study, all selected lines underwent six seasons of testing at ICRISAT. Based on their performance during this period, the germplasm was shared with partners for evaluation at their respective locations. As a result, the tested germplasm is well adapted to the ICRISAT environment, although it may not fully represent the diversity of all target populations of environments (TPEs). In 2021, ICRISAT adopted a revised multi-environment testing strategy aimed at maximizing genetic gain by leveraging specific adaptation to fully realize genetic potential. Since then, groundnut lines have been tested exclusively within their target TPEs, with no agronomic performance evaluations conducted outside these environments. For traits such as HSW and SP, the genetic gain trend did not show a consistent trend, thus highlighting the need for extensive ERA trials with more lines from each cohort to understand the patterns of genetic improvement.

This study primarily used ERA trials to estimate RGG across years and seasons. Although historical data are available at ICRISAT, it could not be utilized due to the absence of consistent check connectivity, which is essential for meaningful comparisons. The absence of consistent checks or benchmarks over time further complicates the estimation of RGG (Rutkoski, 2019a; Covarrubias-Pazaran, 2020). Limited replication of certain genotypes and incomplete historical data also reduce the precision of genetic gain estimates. Therefore, to use historical data, it is recommended to establish a robust strategy for maintaining check continuity across years, which ICRISAT has adopted recently under the revised multi-environment testing strategy. G × E effects in ERA trials can be mitigated by employing the estimated breeding value (EBV) method, which connects time-disjointed data using additive relationship matrices (pedigree- or marker-based). While this accounts for relatedness across years, care must be taken as heritability-dependent shrinkage may underestimate RGG. Alternatively, explicitly designed ERA trials with overlapping time-window entries can improve connectivity and help disentangle year effects from true genetic trends (Piepho et al., 2014). BLUP value can be analyzed and considered in future research. Furthermore, ERA trials often emphasize yield traits, potentially overlooking gains or trade-offs in other important but less-measured traits like quality, stress tolerance, or shelling percentage (Rutkoski, 2019b). These limitations highlight the need for cautious interpretation of ERA trial results and emphasize the importance of integrating genomic tools and standardized protocols to enhance the reliability of genetic gain assessment. Moreover, changes in trait priorities, selection intensity, and breeding objectives across breeding cycles may bias comparisons.

### 4.6 Way forward

To effectively estimate realized genetic gain, the breeding programs must implement a robust check strategy that ensures check continuity across years and environments. This involves the consistent inclusion of common check varieties in multi-year trials, creating a check continuum that enables meaningful comparisons over time. Such an approach is essential for leveraging historical trial data, allowing breeders to accurately assess genetic progress and identify trends in trait improvement. Establishing and maintaining this check framework will enhance the program’s ability to monitor genetic gain and guide future breeding decisions with greater precision. Insights from RGG are invaluable for shaping and refining future breeding strategies. It quantifies how much genetic improvement has been achieved for key traits over time and identifies which traits or germplasm groups are responding well to selection and which are lagging. This helps assess the efficiency of current selection methods, population development, and resource allocation. In the present study, it was identified that progress was made for pod yield with substantial genetic gain, but progress is lagging for the SP trait among the traits studied, and among the germplasm groups, the early-maturing germplasm group is lagging.

Selection for surrogate traits that are closely linked to yield, particularly those with high heritability and more predictable selection responses, can enhance breeding efficiency (Varshney et al., 2013; Snowdon et al., 2021; Wang et al., 2021). Among these key traits studied, HSW has shown the maximum realized genetic gain of 0.44% per annum, indicating scope to target this trait for indirectly improving PY. The high heritability and genetic advancement associated with HSW further support its use as a surrogate trait for PY. In contrast, although SP is an important yield-attributing trait, its utility as a surrogate trait is limited due to strong environmental influence (Gelaye and Luo, 2024; Kona et al., 2024). Moreover, the factors such as pod structure and seed size, which influence SP, may interact antagonistically with other yield components, complicating selection (Bauchet et al., 2019; Cooper et al., 2014). Moreover, through accurate phenotyping for SP, a better understanding of the genetic factors underlying the limited GG in SP will be crucial to guide the design of breeding strategies for improving shelling outturn in groundnut. A deeper understanding of the genetic and physiological bases of SP is needed to target genetic improvement of SP. Further dissecting the genetic architecture of SP using quantitative trait loci (QTL) mapping and genome-wide association studies (GWAS) can pinpoint specific genomic regions associated with this trait.

In groundnut, PY, HSW, and SP are complex traits; hence, to achieve accelerated cultivar development, the way forward lies in leveraging genomic selection (GS) models, which utilize genome-wide markers to predict breeding values and have demonstrated strong potential to enhance genetic gain for complex traits (Mousavi-Derazmahalleh et al., 2019; Anilkumar et al., 2022; Lubanga et al., 2024). Integrating GS into the breeding program can facilitate the identification of favorable genetic combinations that enhance shelling percentage, hundred-kernel weight, and other yield attributes, ultimately improving pod yield. Improving the precision of selection for SP through GS could be pivotal (Gaynor et al., 2017; Khedikar et al., 2018). Re-evaluating breeding strategies to incorporate multi-trait selection indices that balance shelling percentage with other yield components can offer significant advantages (Rani et al., 2024).

## 5 Conclusion

The ICRISAT breeding program has achieved realized genetic gains in pod yield of 1.62% (27.1 kg ha^-^¹ year^-^¹) and 1.52% (25.32 kg ha^-^¹ year^-^¹) for medium- and late-maturing groundnut germplasm, respectively, indicating effective yield improvement in these maturity groups. In contrast, early-maturing germplasm showed a lower realized genetic gain of 0.5% (8.37 kg ha^-^¹ year^-^¹), highlighting the need for targeted breeding strategies focusing on traits such as early vigor, photosynthetic efficiency, and source–sink dynamics to boost yield in this group. Yield improvements, particularly in PY and HSW, were more pronounced during the rainy season, suggesting that current breeding strategies are better aligned with rainy-season conditions. However, the relatively low genetic gains for SP and HSW point to the potential for further enhancement of these traits to drive greater gains in pod yield. The realized genetic gain for shelling outturn, a critical trait for both market value and productivity, has remained low across maturity groups, market types, and seasons. To improve genetic gain for this trait, breeding strategies must include targeted selection for shelling percentage and a deeper understanding of the genetic basis of shell thickness. At ICRISAT, CT has been integrated into the breeding pipeline to enable precise measurement of SP. In parallel, genomic selection models are being developed to support more efficient selection for these traits along with other nutritional, biotic, and abiotic stress resistance traits. A holistic approach combining genomic selection, advanced phenotyping, data analytics, machine learning for extracting insights from large datasets, and simulation-based optimization of selection strategies is essential for accelerating cultivar development.

## Data Availability

The original contributions presented in the study are included in the article/**Supplementary Material**. Further inquiries can be directed to the corresponding author.
