# Supplementary material for: Realized genetic gain for yield and yield attributes in groundnut breeding at ICRISAT from an ERA trial
Source: Front Plant Sci. 2025 Sep 17;16:1640041. doi: 10.3389/fpls.2025.1640041 (PMC12484242; doi:10.3389/fpls.2025.1640041)
Supplement: Supplementary file 1 [file Table1.docx]

Supplementary Table.1 Year wise advancements of genotypes in EYTGG-PC-1 Trial

| SI | Year | Number of entries | Genotypes |
| --- | --- | --- | --- |
| 1 | 1993 | 2 | ICGV 91114 |
| 2 |  |  | ICGV 91116 |
| 3 | 1994 | 2 | ICGV 92195 |
| 4 |  |  | ICGV 92209 |
| 5 | 1995 | 1 | ICGV 93382 |
| 6 | 1996 | 2 | ICGV 94299 |
| 7 |  |  | ICGV 94341 |
| 8 | 1997 | 1 | ICGV 95245 |
| 9 | 1998 | 2 | ICGV 96333 |
| 10 |  |  | ICGV 96390 |
| 11 | 1999 | 3 | ICGV 97243 |
| 12 |  |  | ICGV 97245 |
| 13 |  |  | ICGV 97262 |
| 14 | 2001 | 1 | ICGV 99206 |
| 15 | 2002 | 2 | ICGV 00290 |
| 16 |  |  | ICGV 00308 |
| 17 | 2003 | 1 | ICGV 01020 |
| 18 | 2004 | 3 | ICGV 02022 |
| 19 |  |  | ICGV 02038 |
| 20 |  |  | ICGV 02144 |
| 21 | 2005 | 3 | ICGV 03169 |
| 22 |  |  | ICGV 03194 |
| 23 |  |  | ICGV 03207 |
| 24 | 2006 | 1 | ICGV 04017 |
| 25 | 2008 | 2 | ICGV 06237 |
| 26 |  |  | ICGV 06285 |
| 27 | 2009 | 2 | ICGV 07210 |
| 28 |  |  | ICGV 07217 |
| 29 | 2011 | 2 | ICGV 09002 |
| 30 |  |  | ICGV 09014 |
| 31 | 2012 | 2 | ICGV 10001 |
| 32 |  |  | ICGV 10021 |
| 33 | 2015 | 3 | ICGV 13027 |
| 34 |  |  | ICGV 13189 |
| 35 |  |  | ICGV 13229 |
| 36 | 2016 | 2 | ICGV 14410 |
| 37 |  |  | ICGV 14421 |
| 38 | 2017 | 2 | ICGV 15284 |
| 39 |  |  | ICGV 15290 |
| 40 |  |  | JL 24 (C) |
| 41 |  |  | TMV 2 (C) |

C- Check line

Supplementary Table.2 Year wise advancements of genotypes in EYTGG-SB-PC-2 Trial

| SI | Year | Number of entries | Genotypes |
| --- | --- | --- | --- |
| 1 | 1988 | 1 | ICGV 86590 |
| 2 | 1993 | 1 | ICGV 91223 |
| 3 | 1994 | 4 | ICGV 92035 |
| 4 |  |  | ICGV 92050 |
| 5 |  |  | ICGV 92097 |
| 6 |  |  | ICGV 92121 |
| 7 | 1995 | 4 | ICGV 93128 |
| 8 |  |  | ICGV 93233 |
| 9 |  |  | ICGV 93260 |
| 10 |  |  | ICGV 93261 |
| 11 | 1996 | 1 | ICGV 94118 |
| 12 | 1997 | 1 | ICGV 95066 |
| 13 | 1998 | 1 | ICGV 96174 |
| 14 | 1999 | 1 | ICGV 97087 |
| 15 | 2000 | 1 | ICGV 98077 |
| 16 | 2001 | 2 | ICGV 99019 |
| 17 |  |  | ICGV 99241 |
| 18 | 2002 | 5 | ICGV 00162 |
| 19 |  |  | ICGV 00187 |
| 20 |  |  | ICGV 00211 |
| 21 |  |  | ICGV 00213 |
| 22 |  |  | ICGV 00351 |
| 23 | 2003 | 3 | ICGV 01260 |
| 24 |  |  | ICGV 01265 |
| 25 |  |  | ICGV 01279 |
| 26 | 2005 | 3 | ICGV 03042 |
| 27 |  |  | ICGV 03043 |
| 28 |  |  | ICGV 03057 |
| 29 | 2007 | 1 | ICGV 05155 |
| 30 | 2008 | 5 | ICGV 06039 |
| 31 |  |  | ICGV 06040 |
| 32 |  |  | ICGV 06146 |
| 33 |  |  | ICGV 06420 |
| 34 |  |  | ICGV 06424 |
| 35 | 2009 | 4 | ICGV 07010 |
| 36 |  |  | ICGV 07120 |
| 37 |  |  | ICGV 07220 |
| 38 |  |  | ICGV 07222 |
| 39 | 2012 | 2 | ICGV 10143 |
| 40 |  |  | ICGV 10178 |
| 41 | 2013 | 4 | ICGV 11380 |
| 42 |  |  | ICGV 11396 |
| 43 |  |  | ICGV 11418 |
| 44 |  |  | ICGV 11422 |
| 45 | 2015 | 3 | ICGV 13254 |
| 46 |  |  | ICGV 13265 |
| 47 |  |  | ICGV 13317 |
| 48 | 2016 | 2 | ICGV 14001 |
| 49 |  |  | ICGV 14030 |
| 50 | 2017 | 4 | ICGV 15019 |
| 51 |  |  | ICGV 15073 |
| 52 |  |  | ICGV 15074 |
| 53 |  |  | ICGV 15083 |

Supplementary Table.3 Year wise advancements of genotypes in EYTGG-VB-PC-2 Trial

| SI | Year | Number of entries | Genotypes |
| --- | --- | --- | --- |
| 1 | 1994 | 1 | ICGV 92054 |
| 2 | 1995 | 1 | ICGV 93162 |
| 3 | 1997 | 1 | ICGV 95111 |
| 4 | 1998 | 2 | ICGV 96165 |
| 5 |  |  | ICGV 96266 |
| 6 | 1999 | 2 | ICGV 97115 |
| 7 |  |  | ICGV 97150 |
| 8 | 2000 | 2 | ICGV 98184 |
| 9 |  |  | ICGV 98385 |
| 10 | 2002 | 2 | ICGV 00064 |
| 11 |  |  | ICGV 00246 |
| 12 | 2003 | 1 | ICGV 01491 |
| 13 | 2005 | 1 | ICGV 03287 |
| 14 | 2007 | 1 | ICGV 05057 |
| 15 | 2008 | 1 | ICGV 06175 |
| 16 | 2009 | 2 | ICGV 07247 |
| 17 |  |  | ICGV 07262 |
| 18 | 2012 | 3 | ICGV 10371 |
| 19 |  |  | ICGV 10373 |
| 20 |  |  | ICGV 10379 |
| 21 | 2016 | 2 | ICGV 14224 |
| 22 |  |  | ICGV 14232 |
| 23 | 2017 | 1 | ICGV 15094 |
| 24 |  |  | ICGV 86699 (C) |
| 25 |  |  | ICGV 87846 (C) |

C- Check line

Supplementary Table.4 Year wise advancements of genotypes in EYTGG-SB-PC-3 Trial

| SI | Year | Number of entries | Genotypes |
| --- | --- | --- | --- |
| 1 | 1995 | 1 | ICGV 93058 |
| 2 | 1996 | 1 | ICGV 94204 |
| 3 | 1997 | 1 | ICGV 95163 |
| 4 | 1998 | 2 | ICGV 96066 |
| 5 |  |  | ICGV 96073 |
| 6 | 1999 | 2 | ICGV 97040 |
| 7 |  |  | ICGV 97045 |
| 8 | 2000 | 1 | ICGV 98412 |
| 9 | 2001 | 1 | ICGV 99105 |
| 10 | 2002 | 1 | ICGV 00380 |
| 11 | 2003 | 1 | ICGV 01369 |
| 12 | 2004 | 1 | ICGV 02229 |
| 13 | 2005 | 1 | ICGV 03137 |
| 14 | 2007 | 2 | ICGV 05170 |
| 15 |  |  | ICGV 05182 |
| 16 | 2008 | 3 | ICGV 06188 |
| 17 |  |  | ICGV 06189 |
| 18 |  |  | ICGV 06211 |
| 19 | 2012 | 3 | ICGV 10200 |
| 20 |  |  | ICGV 10209 |
| 21 |  |  | ICGV 10213 |
| 22 | 2013 | 3 | ICGV 11310 |
| 23 |  |  | ICGV 11321 |
| 24 |  |  | ICGV 11353 |
| 25 | 2014 | 2 | ICGV 12266 |
| 26 |  |  | ICGV 12270 |
| 27 |  |  | TKE-19-A (C) |
| 28 |  |  | TPG 41 (C) |

C- Check line

Supplementary Table.5 Year wise advancements of genotypes in EYTGG-VB-PC-3 Trial

| SI | Year | Number of entries | Genotypes |
| --- | --- | --- | --- |
| 1 | 1994 | 1 | ICGV 92160 |
| 2 | 1996 | 1 | ICGV 94215 |
| 3 | 1997 | 1 | ICGV 95165 |
| 4 | 2000 | 1 | ICGV 98432 |
| 5 | 2002 | 2 | ICGV 00440 |
| 6 |  |  | ICGV 00441 |
| 7 | 2003 | 1 | ICGV 01457 |
| 8 | 2004 | 1 | ICGV 02249 |
| 9 | 2005 | 1 | ICGV 03133 |
| 10 | 2007 | 1 | ICGV 05200 |
| 11 | 2008 | 2 | ICGV 06214 |
| 12 |  |  | ICGV 06229 |
| 13 | 2012 | 2 | ICGV 10237 |
| 14 |  |  | ICGV 10272 |
| 15 | 2014 | 1 | ICGV 12218 |
| 16 |  |  | ICGV 86564 (C) |

C- Check line
